# A quantitative synthesis study on body mass index and associated factors among adult men and women in Switzerland

**DOI:** 10.1017/jns.2022.66

**Published:** 2022-08-10

**Authors:** Katarina L. Matthes, Christina Hartmann, Michael Siegrist, Michel Burnier, Murielle Bochud, Marcel Zwahlen, Nicole Bender, Kaspar Staub

**Affiliations:** 1 Institute of Evolutionary Medicine, University of Zurich, Winterthurerstrasse 190, CH-8057 Zurich, Switzerland; 2 Department of Health Sciences and Technology (D-HEST), Consumer Behavior, ETH Zurich, Zurich, Switzerland; 3 Service of Nephrology and Hypertension, Lausanne University Hospital, Lausanne, Switzerland; 4 Center for Primary Care and Public Health (Unisanté), University of Lausanne, Lausanne, Switzerland; 5 Institute of Social and Preventive Medicine, University of Bern, Bern, Switzerland; 6 Zurich Center for Integrative Human Physiology (ZIHP), University of Zurich, Zurich, Switzerland

**Keywords:** Body mass index, Excess weight, Lifestyle factors, Obesity, Synthesis study

## Abstract

Excess weight is caused by multiple factors and has increased sharply in Switzerland since the 1990s. Its consequences represent a major challenge for Switzerland, both in terms of health and the economy. Until now, there has been no cross-dataset overview study on excess weight in adults in Switzerland. Therefore, our aim was to conduct the first synthesis on excess weight in Switzerland. We included all existing nationwide Swiss studies (eight total), which included information on body mass index (BMI). Mixed multinomial logistic regression analyses were performed to assess the associations between different socio-demographic, lifestyle cofactors and the World Health Organization (WHO) categories for BMI. Along with lifestyle factors, socio-demographic factors were among the strongest determinants of BMI. In addition, self-rated health status was significantly lower for underweight, pre-obese and obese men and women than for normal weight persons. The present study is the first to synthesise all nationwide evidence on the importance of several socio-demographic and lifestyle factors as risk factors for excess weight. In particular, the highlighted importance of lifestyle factors for excess weight opens up the opportunity for further public health interventions.

## Introduction

Excess weight (BMI ≥ 25⋅0 kg/m^2^) has increased sharply in Switzerland since the 1990s, and its consequences represent a major challenge for Switzerland, both in terms of health and the economy^(1)^. In 2007, it was estimated that 27 000 cases of type 2 diabetes, 63 000 cases of high blood pressure and 37 000 cases of dyslipidaemia may have been avoided had overweight and obesity in Switzerland been maintained at the 1992 level^(2)^. Additionally, the direct and indirect costs of excess weight have tripled between 2002 and 2012, amounting to approximately 8⋅0 billion Swiss francs^(1)^.

It has widely been shown that excess weight has multifactorial explanations^(3)^. In westernised countries such as Switzerland, factors such as sex, age, socio-demographic background, educational and lifestyle factors, including dietary habits, physical activity levels, alcohol consumption, sleep health and tobacco use, have been shown to be associated with excess weight^(4–7)^. A less common health problem in westernised countries is underweight. However, being underweight is also associated with poor self-rated health and increased mortality^(8–11)^.

Studies from the annual weight monitoring of conscripts^(12)^ and school children^(13)^ have shown that the prevalence of excess weight, at least among young people, has not increased since approximately 2010. For the adult population, the prevalence of excess weight varies between 25 and 50 %, depending on the study, reflecting differences in the design and the composition of the study population in terms of sex, age and socio-economic and ethnic backgrounds. Recent studies have focused mainly on individual datasets^(14)^, some of which have a regional focus^(15)^. However, the trend in research is moving towards synthesis or pooled studies, which have been performed very successfully at the global scale for several years^(16)^.

In Switzerland, existing nationwide adult studies on body mass index (BMI) have recently been analysed individually but never synthesised. The most recent comparison study was an overview in German, reported mostly as a narrative and performed more than 5 years ago^(17)^. To date, no quantitative synthesis study of eight individual studies has been performed to analyse the association between BMI and different socio-demographic and lifestyle factors. Closing this research gap would allow public health experts to identify risk factors for excess- and underweight in the adult population and associated factors with greater precision. Eventually, this research would allow intervention programmes to be tailored more appropriately.

Our goal was thus to conduct the first synthesis study on excess weight in Switzerland. We identified and accessed all existing nationwide studies from areas of health, nutrition and economics, which also included measured or self-reported information on body weight and height. We investigated whether explanatory factors in the areas of socio-demographics, nutrition, exercise or lifestyle were available in the included studies and how these factors were associated with BMI. To our knowledge, this is the first synthesis study performed with data on a single country.

## Materials and methods

### Data

For our analysis, we included eight national Swiss studies with information on BMI and potential risk factors:

#### menuCH

menuCH is a representative Swiss national nutrition survey conducted between 2014 and 2015. A total of 2057 participants were included in the analysis. Body height and weight were available in both measured and self-reported form. Since the measured data are more accurate, these have been used in the analysis. A more detailed description of the data collection, recruitment procedure and participation rate was published elsewhere^(18)^.

#### Swiss Health Survey

The Swiss Health Survey (SHS) is a representative survey of health conditions, health behaviour and the use of health services by people living in Switzerland and has been conducted every 5 years since 1992. In the present study, we included the 2012 (21 597 participants) and 2017 (22 134 participants) surveys. Both surveys were analysed separately. Self-reported body height and weight were asked in structured telephone interviews. A more detailed description of the data collection, recruitment procedure, participant rate and sample weight strategy was published elsewhere^(19,20)^.

#### Swiss Household Panel

The Swiss Household Panel (SHP) is a representative longitudinal survey on social changes and living conditions of the population in Switzerland and has been conducted every year since 1999 with three cohorts (starting in 1999, 2004 and 2013). In the present study, cross-sectional data on the third cohort (staring 2013) consisting of 5040 participants and obtained in 2014 were used. Self-reported body height and weight were asked to all household members aged 15 years and over in individual telephone interviews. A more detailed description of the data collection, recruitment procedure, participant rate and sample weight strategy was published elsewhere^(21)^.

#### Statistics on Income and Living Conditions

The Swiss survey on Statistics on Income and Living Conditions (SILC) is a representative study on income and living conditions of households in Switzerland. Households and their members are surveyed for several years, and new households are added each year. In the present study, cross-sectional data on a total of 12 980 participants obtained in 2017 were used. Self-reported body height and weight were asked to household members aged 16 years and over in structured telephone interviews. A more detailed description of the data collection, recruitment procedure, participant rate and sample weight strategy was published elsewhere^(22)^.

#### Swiss Food Panel

The Swiss Food Panel (SFP) is a longitudinal survey about Swiss eating behaviours. SFP 1⋅0 was conducted between 2010 and 2014, and SFP 2⋅0 was initiated in 2017. For the present study, only cross-sectional data from the 2010 survey, SFP 1⋅0, and from the 2017 survey, SFP 2⋅0, were used. In 2010, a total of 6161 participants were included, and in 2017, a total of 5587 participants were included. Survey from both years were analysed separately. Self-reported body height and weight were asked in structured questionnaires. A more detailed description of the data collection, recruitment procedure and participant rate was published elsewhere^(23,24)^.

#### Swiss Survey on Salt

The Swiss Survey on Salt (SSS) was conducted in 2010 and 2011 with 1539 participants to estimate the mean dietary salt intake of people living in Switzerland. Body height and weight were available measured form. A more detailed description of the data collection, recruitment procedure and participant rate was published elsewhere^(25)^.

### Body mass index

We used participant self-reported body height and body weight or measured body height weight (menuCH and SSS), respectively, to calculate the BMI (kg/m^2^). Participants with a BMI between 14⋅0 and 60⋅0 kg/m^2^ were included. BMI was categorised using the WHO classification^(26)^: underweight (BMI < 18⋅5 kg/m^2^), normal weight (18⋅5 BMI < 25⋅0 kg/m^2^), pre-obesity (25⋅0 BMI < 30⋅0 kg/m^2^) and obesity (BMI ≥ 30⋅0 kg/m^2^).

### Socio-demographic and lifestyle factors

In all studies, information on sex, age, language region, education and physical activity was available. The three main language regions, namely German-, French- and Italian-speaking regions, were defined by the Swiss Federal Office of Statistics^(27)^. Education was classified into three categories: primary (no degree or a compulsory school degree), secondary (completed high school or apprenticeship) and tertiary (higher degree for which a high school diploma was required). Since all studies assessed physical activity with different methods, questions and units (SFP 2017, very light active to very active; SSS, nearly never to more than two times per week; SILC, minutes per week; SFP 2010, SHS, SHP and menuCH, 0–7 days per week), standardised *z*-scores were calculated to make physical activity comparable between the studies.

Further variables were available only for some studies. Therefore, additional analyses were performed with subsets of these studies. Using binary coding, urbanity (available in 5/8 studies) was coded as either ‘urban’ or ‘rural’^(28)^, and nationality (available in 7/8 studies) was coded as either ‘Swiss’ or ‘Non-Swiss’. Smoking (available in 5/8 studies) was coded as ‘yes’ (current smoker) or ‘no’ (non-smoker)’. If physical activity, measured in hours per week, was available in addition to physical activity (available in 3/8 studies), we categorised it according to the Swiss physical activity recommendations for adults, which recommends at least 2⋅5 h of exercise per week^(29)^. Less than 2⋅5 h of activity was defined as ‘not fulfilled: <2⋅5 h’, and 2⋅5 h of activity or more was defined as ‘yes/fulfilled: ≥2⋅5 h’. The self-rated health status (available in 7/8 studies) was categorised as ‘very good’, ‘good’, ‘average’ and ‘poor and very poor’. The last group was combined because of small sample size. Information on self-reported sleep disorders were available only for the SHS (2012 and 2017) (available in 2/8 studies) and was categorised as ‘none or little’, ‘some’ and ‘pathological’. The data on fruit and vegetable, meat, fish, milk and dairy, sweetened beverages and alcohol consumption were *z*-transformed because they were assessed with different methods, questions and units (e.g. SFP, seldom/never, several times a year/month/week to daily; SSS, portions/day: never, <1, 1–2, 3–4 or >5; SILC, at least 2 times per day, once per day, 4–6 times per day, 1–3 times per week, less than once per week, never; SHS, days/week: never, ≤1, 2–3, 4–5, 6 days or daily; and menuCH, intake was recorded in grams).

### Statistical analyses

Mixed multinomial logistic regression analyses were used to assess the associations between the different socio-demographic and lifestyle factors and WHO categories for BMI (normal weight was the reference category). With this model, we did not pool the studies, but included the eight discrete studies as random factor to control for variance within and between the eight discrete studies.

When available, sample weights of the respective studies were considered in the analysis. We stratified all models by sex and adjusted for age, language region, education, body height and physical activity (except for the model using the recommendation for physical activity, in which we excluded physical activity as a covariate in *z*-scores). In addition, we visualised the probabilities of being in a one BMI group given some specific predictors.

To examine the influence of each independent variable on BMI, we removed each independent variable from the model one at a time and calculated the Akaike information criterion (AIC). Next, the difference between the AIC for the full model *M* and the model with the omission of an independent variable *k* was calculated, i.e. ΔAIC*_k_* = AIC*_k_* − AIC*_M_*. The larger ΔAIC*_k_* is, the more important the variable in the model.

All statistical

analyses were performed using R Version 4.0.5 software^(30)^. The R package ‘lme4’^(31)^ was used to calculate the mixed models and ‘ggplot2’^(32^) was used to produce the figures.

## Results

Table 1 shows the descriptive characteristics of each study stratified by sex. The proportion of participants who were obese was higher in the SSS for men and women than in the other studies. The participants in both SFPs were older than those in other studies, especially men. In the SSS, the German-speaking language region was underrepresented, whereas the Italian-speaking language region was overrepresented. The proportion of high education was highest in the menuCH and SFP studies. In the menuCH study, two-thirds of the participants stated that they performed less than 2⋅5 h per week of physical activity. In contrast, participants of the SILC and SFP 2010 study stated the opposite; namely, 80 % performed more than 2⋅5 h per week of physical activity. The self-rated health status was remarkably poorer in both SFP studies than in the other studies; approximately 60 % rated their health status as ‘average’ or ‘poor or very poor’, whereas in the other studies, the percentages in these categories were remarkably lower.

**Table 1.** Characteristics of each study

	Men								Women							
	menuCH^a^	SHS 2012^a^	SHS 2017^a^	SHP^a^	SILC^a^	SFP 2010	SFP 2017	SSS	menuCH^a^	SHS 2012^a^	SHS 2017^a^	SHP^a^	SILC^a^	SFP 2010	SFP 2017	SSS
	%	%	%	%	%	%	%	%	%	%	%	%	%	%	%	%
BMI																
Underweight: <18⋅5 kg/m^2^	0⋅8	0⋅7	0⋅8	0⋅4	0⋅8	0⋅5	0⋅5	1⋅0	3⋅8	5⋅7	4⋅8	5⋅5	4⋅5	5⋅5	4⋅2	4⋅7
Normal weight: 18⋅5–24⋅9 kg/m^2^	42⋅4	46⋅9	46⋅4	48⋅0	45⋅5	44⋅7	45⋅3	42⋅3	65⋅4	60⋅9	60⋅7	60⋅7	60⋅9	66⋅0	67⋅5	56⋅7
Pre-obesity: 25–29⋅9 kg/m^2^	42⋅6	40⋅8	40⋅0	39⋅8	41⋅9	42⋅1	43⋅4	39⋅3	20⋅4	23⋅9	24⋅0	22⋅6	23⋅8	19⋅9	20⋅9	26⋅4
Obesity: >29⋅9 kg/m^2^	14⋅2	11⋅6	12⋅8	11⋅8	11⋅8	12⋅7	10⋅9	17⋅4	10⋅4	9⋅5	10⋅5	11⋅1	10⋅8	8⋅6	7⋅4	12⋅2
Age																
Mean (sd)	46⋅3 (15⋅6)	47⋅5 (17⋅6)	48⋅5 (17⋅7)	47⋅7 (17⋅6)	48⋅2 (17⋅4)	58⋅7 (16⋅7)	56⋅7 (15⋅0)	48⋅5 (18⋅0)	45⋅2 (15⋅2)	49⋅3 (18⋅4)	49⋅7 (18⋅3)	48⋅9 (18⋅5)	49⋅0 (17⋅8)	53⋅5 (16⋅9)	51⋅5 (14⋅8)	46⋅6 (17⋅7)
Language regions																
German	70⋅2	72⋅4	71⋅7	69⋅3	71⋅8	74⋅7	73⋅5	54⋅7	67⋅4	70⋅2	70⋅8	67⋅8	70⋅7	70⋅9	67⋅9	54⋅5
French	24⋅3	23⋅3	23⋅6	23⋅8	23⋅9	25⋅3	26⋅5	31⋅1	27⋅0	24⋅9	24⋅7	26⋅2	24⋅6	29⋅1	32⋅1	31⋅7
Italian	5⋅5	4⋅3	4⋅7	6⋅9	4⋅3	–	–	14⋅2	5⋅6	4⋅9	4⋅5	6⋅0	4⋅7	–	–	13⋅8
Education																
Primary	5⋅0	12⋅6	11⋅4	11⋅5	12⋅4	6⋅3	8⋅1	13⋅2	4⋅3	17⋅4	15⋅3	14⋅8	18⋅3	8⋅1	10⋅2	15⋅0
Secondary	39⋅9	50⋅2	47⋅5	51⋅4	49⋅9	36⋅5	35⋅3	39⋅7	46⋅2	59⋅9	55⋅6	58⋅5	52⋅2	41⋅3	41⋅7	49⋅0
Tertiary	55⋅1	37⋅2	41⋅1	37⋅1	37⋅7	57⋅2	56⋅7	47⋅1	49⋅5	22⋅7	29⋅1	26⋅8	29⋅5	50⋅6	48⋅1	36⋅0
Body height in cm																
Mean (sd)	176⋅4 (7⋅5)	176⋅9 (7⋅2)	177⋅4 (7⋅2)	177⋅0 (7⋅0)	177⋅4 (6⋅9)	176⋅7 (6⋅9)	176⋅6 (7⋅0)	176⋅2 (7⋅0)	164⋅4 (7⋅0)	164⋅6 (6⋅5)	164⋅6 (6⋅6)	164⋅7 (6⋅6)	164⋅7 (6⋅6)	165⋅3 (6⋅2)	165⋅4 (6⋅1)	164⋅5 (6⋅7)
Physical activity	−1⋅1 to 1⋅9	−1⋅2 to 1⋅7	−1⋅3 to 1⋅6	−1⋅2 to 1⋅9	−0⋅9 to 9⋅7	−2⋅2 to 1⋅2	−2⋅3 to 2⋅3	−1⋅7 to 1⋅1	−1⋅1 to 1⋅9	−1⋅2 to 1⋅7	−1⋅3 to 1⋅6	−1⋅2 to 1⋅9	−0⋅9 to 14⋅0	−2⋅2 to 1⋅2	−2⋅3 to 2⋅3	−1⋅7 to 1⋅1
Urbanity																
Urban	52⋅6	73⋅0	73⋅6	–	–	42⋅8	46⋅9	–	56⋅6	73⋅8	74⋅7	–	–	49⋅2	49⋅4	–
Rural	47⋅4	27⋅0	26⋅4	–	–	57⋅2	53⋅1	–	43⋅4	26⋅2	25⋅3	–	–	50⋅8	50⋅6	–
Nationality																
Swiss	73⋅5	75⋅2	74⋅6	74⋅1	73⋅6	84⋅3	–	84⋅0	74⋅5	78⋅9	78⋅0	76⋅8	76⋅9	81⋅8	–	89⋅3
Non-Swiss	26⋅5	24⋅8	25⋅4	25⋅9	26⋅4	15⋅7	–	16⋅0	25⋅5	21⋅1	22⋅0	23⋅2	23⋅1	18⋅2	–	10⋅7
Smoking																
No	70⋅5	67⋅9	68⋅7	75⋅1	–	–	–	81⋅5	84⋅3	75⋅9	76⋅5	79⋅8	–	–	–	83⋅8
Yes	29⋅5	32⋅1	31⋅3	24⋅9	–	–	–	18⋅5	15⋅7	24⋅1	23⋅5	20⋅2	–	–	–	16⋅2
Fruit and Vegetable consumption^b^	−6⋅1 to 2⋅4	−4⋅2 to 0⋅8	−4⋅2 to 0⋅8	–	−3⋅3 to 3⋅4	−3⋅6 to 1⋅4	−3⋅9 to 3⋅5	−3⋅3 to 3⋅4	−6⋅1 to 2⋅2	−4⋅2 to 0⋅8	−4⋅2 to 0⋅8	–	−3⋅3 to 3⋅4	−4⋅2 to 0⋅8	−4⋅2 to 0⋅8	−3⋅3 to 3⋅4
Meat consumption^b^	−2⋅4 to 1⋅4	−2⋅5 to 1⋅5	−2⋅3 to 1⋅5	–	–	−2⋅7 to 4⋅2	−1⋅8 to 7⋅1	−2⋅2 to 1⋅7	−2⋅4 to 1⋅2	−2⋅5 to 1⋅5	−2⋅3 to 1⋅5	–	–	−2⋅7 to 3⋅5	−1⋅8 to 6⋅0	−2⋅2 to 1⋅7
Fish consumption^b^	−0⋅7 to 2⋅4	−1⋅7 to 5⋅6	−1⋅6 to 5⋅3	–	–	−2⋅3 to 4⋅0	−1⋅3 to 6⋅4	−1⋅6 to 5⋅8	−0⋅7 to 2⋅3	−1⋅7 to 5⋅6	−1⋅6 to 5⋅3	–	–	−2⋅3 to 4⋅0	−1⋅3 to 6⋅4	−1⋅6 to 5⋅8
Milk and dairy consumption^b^	−4⋅0 to 2⋅1	−2⋅7 to 1⋅1	–	–	–	−0⋅9 to 3⋅1	−1⋅7 to 4⋅0	–	−4⋅0 to 1⋅6	−2⋅7 to 1⋅1	–	–	–	−0⋅9 to 3⋅6	−1⋅7 to 3⋅3	–
Sweetened beverages consumption^b^	−1⋅4 to 1⋅7	–	−0⋅8 to 2⋅1	–	–	−0⋅9 to 4⋅8	−0⋅7 to 5⋅0	–	−1⋅4 to 1⋅6	–	−0⋅8 to 2⋅1	–	–	−0⋅9 to 4⋅8	−0⋅7 to 5⋅0	–
Alcohol consumption^b^	−1⋅1 to 1⋅8	−1⋅9 to 1⋅3	−1⋅8 to 1⋅4	–	–	−1⋅1 to 3⋅8	−1⋅6 to 3⋅6	–	−1⋅1 to 1⋅4	−1⋅9 to 1⋅3	−1⋅8 to 1⋅4	–	–	−1⋅1 to 3⋅8	−1⋅6 to 3⋅0	–
Following recommendation of physical activity																
No: <2⋅5 h	64⋅6	–	–	–	22⋅1	22⋅3	–	–	64⋅4	–	–	–	22⋅9	25⋅2	–	–
Yes: ≥2⋅5 h	35⋅4	–	–	–	77⋅9	77⋅7	–	–	35⋅6	–	–	–	77⋅1	74⋅8	–	–
Self-rated health status																
Very good	29⋅5	40⋅1	41⋅4	26⋅0	36⋅5	6⋅7	6⋅7	–	34⋅8	36⋅8	38⋅8	20⋅6	32⋅4	6⋅4	8⋅1	–
Good	57⋅3	44⋅0	44⋅0	62⋅3	44⋅8	33⋅9	32⋅0	–	54⋅8	44⋅2	44⋅3	62⋅5	46⋅2	36⋅2	37⋅1	–
Average	11⋅8	12⋅3	10⋅7	9⋅7	14⋅6	46⋅1	50⋅4	–	9⋅5	15⋅2	13⋅4	14⋅3	17⋅0	47⋅3	46⋅2	–
Poor and very poor	1⋅4	3⋅6	3⋅9	2⋅0	4⋅0	13⋅3	10⋅9	–	0⋅9	3⋅8	3⋅5	2⋅6	4⋅3	10⋅0	8⋅6	–
Sleep disorder																
None or little	–	79⋅6	73⋅8	–	–	–	–	–	–	72⋅4	67⋅9	–	–	–	–	–
Some	–	15⋅8	21⋅2	–	–	–	–	–	–	20⋅3	24⋅3	–	–	–	–	–
Pathological	–	4⋅6	4⋅9	–	–	–	–	–	–	7⋅4	7⋅8	–	–	–	–	–

If a variable does not exist in a dataset, the respective field is empty.

a Weighted.

b Variable is *z*-transformed (Mean = 0, sd = 1), therefore the range of the *z*-scores is shown.

Table 2 presents the multinomial regression results. The findings for men and women were similar. Differences between men and women were mainly observed for the language regions. The risk for pre-obesity *v*. normal weight was lower for men living in the French-speaking region of Switzerland than for men living in the German-speaking region (OR 0⋅94 [95 % CI 0⋅89, 0⋅99]). For women in the Italian-speaking region of Switzerland, the risk of obesity was lower than that of women in the German-speaking region (OR 0⋅74 [95 % CI 0⋅64, 0⋅85]), whereas the risk for being underweight was higher (OR 1⋅66 [95 % CI 1⋅39, 1⋅97]). The older the participants were, the higher their odds for pre-obesity and obesity. The higher the education was, the lower the odds for pre-obesity and obesity. In addition, non-Swiss participants revealed a significantly higher risk of pre-obesity (men, OR  1⋅28 [95 % CI 1⋅20, 1⋅36] and women, OR  1⋅29 [95 % CI 1⋅20, 1⋅39]) and obesity (men, OR  1⋅38 [95 % CI 1⋅26, 1⋅52] and women, OR  1⋅46 [95 % CI 1⋅32, 1⋅61]) than Swiss participants. A higher BMI was not associated with the intake of fruits and vegetables, milk and dairy or fish but was associated with increased meat and sweetened beverage consumption. Moreover, performing more than 2⋅5 h of physical activity per week was associated with a lower risk for pre-obesity (men, OR  0⋅80 [95 % CI 0⋅72, 0⋅89] and women, OR  0⋅79 [95 % CI 0⋅71, 0⋅89]) and obesity (men, OR  0⋅52 [95 % CI 0⋅45, 0⋅61] and women, OR  0⋅59 [95 % CI 0⋅51, 0⋅69]). The odds of self-rated health status were remarkably lower for underweight, pre-obese and obese individuals than for normal weight individuals. Sleep disorders were associated with obesity and being underweight.

**Table 2.** Results of the sex-stratified multinomial logistic regression analyses

	Men			Women		
	Underweight *v*. normal	Pre-obesity *v*. normal	Obesity *v*. normal	Underweight *v*. normal	Pre-obesity *v*. normal	Obesity *v*. normal
Age (eight studies)^a^	**0⋅98(0⋅98–0⋅99)**	**1⋅02(1⋅02–1⋅02)**	**1⋅02(1⋅02–1⋅02)**	0⋅99(0⋅99–1⋅00)	**1⋅02(1⋅02–1⋅02)**	**1⋅02(1⋅02–1⋅02)**
Language region (eight studies)^a^						
German	1⋅00	1⋅00	1⋅00	1⋅00	1⋅00	1⋅00
French	1⋅24(0⋅94–1⋅63)	**0⋅94(0⋅89–0⋅99)**	1⋅00(0⋅92–1⋅08)	**1⋅22(1⋅10–1⋅36)**	1⋅04(0⋅98–1⋅10)	1⋅06(0⋅99–1⋅14)
Italian	0⋅94(0⋅53–1⋅66)	1⋅00(0⋅91–1⋅11)	0⋅96(0⋅82–1⋅12)	**1⋅66(1⋅39–1⋅97)**	**0⋅83(0⋅74–0⋅93)**	**0⋅74(0⋅64–0⋅85)**
Education (eight studies)^a^						
Primary	1⋅00	1⋅00	1⋅00	1⋅00	1⋅00	1⋅00
Secondary	**0⋅62(0⋅44–0⋅87)**	1⋅01(0⋅93–1⋅09)	**0⋅88(0⋅79–0⋅99)**	0⋅86(0⋅74–1⋅01)	**0⋅69(0⋅64–0⋅74)**	**0⋅58(0⋅53–0⋅63)**
Tertiary	**0⋅41(0⋅28–0⋅59)**	**0⋅85(0⋅79–0⋅93)**	**0⋅56(0⋅49–0⋅63)**	0⋅93(0⋅79–1⋅10)	**0⋅47(0⋅44–0⋅51)**	**0⋅34(0⋅30–0⋅38)**
Body height (eight studies)^a^	0⋅99(0⋅97–1⋅01)	0⋅99(0⋅99–1⋅00)	**0⋅98(0⋅98–0⋅99)**	**1⋅02(1⋅01–1⋅03)**	**0⋅98(0⋅98–0⋅98)**	**0⋅96(0⋅95–0⋅96)**
Physical activity (eight studies)^a^	1⋅05(0⋅93–1⋅19)	0⋅98(0⋅96–1⋅01)	**0⋅91(0⋅88–0⋅94)**	0⋅99(0⋅95–1⋅04)	**0⋅95(0⋅93–0⋅98)**	**0⋅92(0⋅89–0⋅95)**
Urbanity (five studies)^b^						
Urbanity	1⋅00	1⋅00	1⋅00	1⋅00	1⋅00	1⋅00
Rural	0⋅76(0⋅55–1⋅04)	**1⋅14(1⋅07–1⋅20)**	**1⋅19(1⋅10–1⋅30)**	0⋅89(0⋅79–1⋅01)	**1⋅10(1⋅03–1⋅17)**	**1⋅13(1⋅03–1⋅24)**
Nationality (seven studies)^c^						
Swiss	1⋅00	1⋅00	1⋅00	1⋅00	1⋅00	1⋅00
Non-Swiss	0⋅81(0⋅57–1⋅15)	**1⋅28(1⋅20–1⋅36)**	**1⋅38(1⋅26–1⋅52)**	0⋅94(0⋅82–1⋅08)	**1⋅29(1⋅20–1⋅39)**	**1⋅46(1⋅32–1⋅61)**
Alcohl consumption (five studies)^d^	0⋅91(0⋅79–1⋅05)	1⋅02(0⋅99–1⋅04)	1⋅02(0⋅98–1⋅06)	0⋅96(0⋅91–1⋅02)	**1⋅04(1⋅01–1⋅07)**	0⋅99(0⋅95–1⋅03)
Smoking (five studies)^e^						
Non-smoker	1⋅00	1⋅00	1⋅00	1⋅00	1⋅00	1⋅00
Smoker	1⋅25(0⋅91–1⋅71)	1⋅01(0⋅95–1⋅08)	0⋅96(0⋅87–1⋅05)	**1⋅17(1⋅03–1⋅33)**	0⋅93(0⋅86–1⋅01)	**0⋅84(0⋅75–0⋅93)**
Fruit and Vegetable consumption (seven studies)^f^	1⋅07(0⋅94–1⋅21)	1⋅01(0⋅99–1⋅04)	0⋅99(0⋅96–1⋅03)	1⋅01(0⋅96–1⋅06)	0⋅99(0⋅96–1⋅01)	1⋅00(0⋅96–1⋅03)
Meat consumption (six studies)^g^	1⋅02(0⋅88–1⋅18)	**1⋅04(1⋅02–1⋅07)**	**1⋅11(1⋅06–1⋅15)**	0⋅91(0⋅86–0⋅96)	**1⋅08(1⋅05–1⋅11)**	**1⋅14(1⋅09–1⋅19)**
Fish consumption (six studies)^h^	0⋅88(0⋅76–1⋅02)	0⋅98(0⋅95–1⋅00)	1⋅00(0⋅96–1⋅04)	1⋅01(0⋅95–1⋅06)	1⋅03(1⋅00–1⋅06)	1⋅01(0⋅97–1⋅06)
Milk and dairy consumption (four studies)^i^	0⋅91(0⋅76–1⋅10)	0⋅99(0⋅95–1⋅02)	0⋅96(0⋅91–1⋅01)	0⋅99(0⋅92–1⋅06)	0⋅99(0⋅95–1⋅03)	**1⋅06(1⋅00–1⋅12)**
Sweetened beverage consumption (four studies)^j^	1⋅12(0⋅94–1⋅33)	**1⋅06(1⋅02–1⋅09)**	**1⋅12(1⋅07–1⋅18)**	1⋅03(0⋅96–1⋅11)	**1⋅04(1⋅00–1⋅08)**	**1⋅11(1⋅05–1⋅17)**
Following recommendation of physical activity (three studies)^k^						
No: <2⋅5 h	1⋅00	1⋅00	1⋅00	1⋅00	1⋅00	1⋅00
Yes: >=2⋅5 h	**0⋅46(0⋅28–0⋅75)**	**0⋅80(0⋅72–0⋅89)**	**0⋅52(0⋅45–0⋅61)**	1⋅07(0⋅85–1⋅34)	**0⋅79(0⋅71–0⋅89)**	**0⋅59(0⋅51–0⋅69)**
Self-rated health status (seven studies)^l^						
Very good	1⋅00	1⋅00	1⋅00	1⋅00	1⋅00	1⋅00
Good	1⋅25(0⋅92–1⋅70)	**1⋅40(1⋅33–1⋅48)**	**2⋅30(2⋅09–2⋅54)**	1⋅01(0⋅90–1⋅13)	**1⋅48(1⋅39–1⋅58)**	**2⋅50(2⋅24–2⋅78)**
Average	**2⋅02(1⋅34–3⋅04)**	**1⋅72(1⋅59–1⋅86)**	**3⋅94(3⋅50–4⋅43)**	**1⋅20(1⋅03–1⋅39)**	**1⋅91(1⋅76–2⋅07)**	**4⋅59(4⋅06–5⋅20)**
Poor and very poor	**7⋅42(4⋅65–11⋅84)**	**1⋅86(1⋅64–2⋅12)**	**6⋅36(5⋅39–7⋅51)**	**2⋅20(1⋅74–2⋅77)**	**2⋅26(1⋅97–2⋅58)**	**8⋅56(7⋅25–10⋅10)**
Sleep disorder (two studies)^m^						
None or little	1⋅00	1⋅00	1⋅00	1⋅00	1⋅00	1⋅00
Some	**1⋅98(1⋅28–3⋅07)**	1⋅08(0⋅99–1⋅18)	**1⋅48(1⋅31–1⋅68)**	**1⋅27(1⋅08–1⋅50)**	1⋅08(0⋅99–1⋅18)	**1⋅17(1⋅03–1⋅33)**
Pathological	1⋅46(0⋅58–3⋅66)	1⋅10(0⋅93–1⋅30)	**1⋅73(1⋅39–2⋅15)**	**1⋅31(1⋅01–1⋅68)**	1⋅07(0⋅93–1⋅23)	**1⋅62(1⋅35–1⋅93)**

^b—j, l, m^Results were adjusted for age, education, language regions, physical activity and body height.

^k^Results were adjusted for age, education, language regions and body height.

a All studies included, results were mutually adjusted for all variables.

b menuCH, SHS, SFP included.

c menuCH, SHS, SHP, SILC,SFP 2010, SSS included.

d menuCH, SHS, SFP included.

e menuCH, SHS, SHP included.

f menuCH, SHS, SILC, SFP, SSS included.

g menuCH, SHS, SFP, SSS included.

h menuCH, SHS, SFP, SSS included.

i menuCH, SHS 2012, SFP included.

j menuCH, SHS 2017, SFP included.

k menuCH, SILC, SFP 2010 included.

l menuCH, SHS, SHP, SILC, SFP included.

m SHS.

Bold values significant results.

**Fig. 1.**
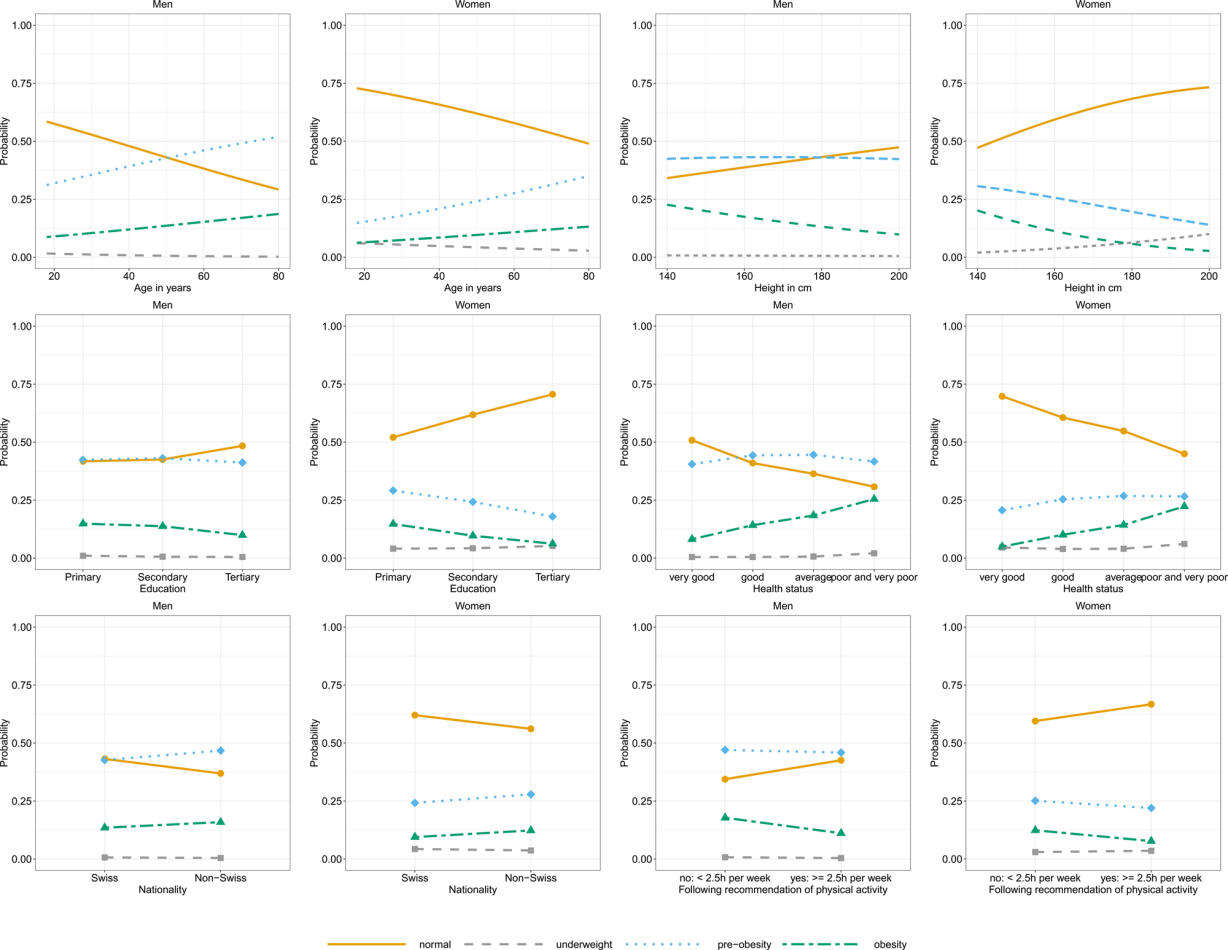
Probabilities being in a specific BMI group given age, height, education, self-rated health status, nationality or recommendation of physical activity stratified.

The probability of being pre-obese or obese increased with higher age, poorer self-rated health status or being non-Swiss and decreased with being taller, having a higher education, or performing more than 2⋅5 h of physical activity per week (Fig. 1). Examining the AIC (Supplementary Table S1), the most important variable to explain BMI was age, followed by education level and body height. Language region and physical activity played less important roles in explaining BMI. Adding the additional explorative variables to the model (nationality, health status and hours spent performing physical activity), it is shown that these were important factors in explaining BMI.

## Discussion

In the present analysis, we compared and synthesised eight national studies, which had information about body height and weight, from Switzerland and analysed the association between BMI and socio-demographic and lifestyle factors. All datasets contained information on BMI, physical activity and socio-economic and language/regional data. Additionally, some datasets contained information on nutrition, alcohol consumption, smoking, self-perceived health or other lifestyle factors. The datasets of these studies differed slightly in the mean age or educational level of the participants, although several studies were designed to be representative of the general population. The SSS and the SFP were not representative, and their participants were, in fact, older (SFP) and showed a higher BMI (SSS) than the participants in the other studies. From the representative studies, menuCH included participants in more urban areas and with higher education than other studies, a fact that might be explained by the challenging assessments used in the study that might have prevented less-educated people from participating (questionnaire, two 24 h dietary recall interviews and anthropometric measurements)^(18)^.

The menuCH study also differed regarding physical activity, with participants performing less physical activity than participants in the SILC and SFP 2010 studies. Physical activity data were collected in different ways. In menuCH, this information was obtained through interview questions, while in the other two studies, the data were collected through a questionnaire. The SILC study asked for physical activity in terms of minutes per week, while SFP 2010 and menuCH asked for physical activity days per week. It is possible that these differences in data collection methods explain the difference in the results and that people answered more accurately when asked in an interview than when completing a questionnaire. However, this point remains speculative and requires more in-depth investigation.

In the two SFP studies, questions about health status were answered more negatively than in the other studies. Notably, the mean age of participants in the SFP studies was higher than that of participants in the other studies. This might explain, at least partially, the poorer self-rated health status, as health status normally correlates with age. However, other factors that might explain this result must not be ignored, as the SFP studies are not representative and might include more people with diseases than the general population.

We confirmed differences in BMI between the sexes and between language regions that had been previously described in publications reporting single studies^(14,33–35)^. BMI differences between the language regions in Switzerland are mainly explained due to different cultural influences in the respective language regions (especially by influences from the neighbouring countries – Germany, France and Italy) and thus to different dietary behaviours^(34)^. In the German-speaking region, for example, milk and dairy, and meat products are consumed more frequently than in the Italian-speaking region^(36)^. However, the pattern that we found can be seen only when all studies are synthesised. For instance, the lower risk for pre-obesity *v*. normal weight in men from the French-speaking regions of Switzerland, compared to that of men from the German-speaking regions, is seen only in the menuCH^(18)^, while the lower risk of obesity for women from the Italian-speaking regions of Switzerland, compared to that of women from the German-speaking region, is seen only in the SSS study^(25)^. Other factors, such as increasing BMI gradients with increasing age or education level, are more established and can be found in all studies, including the two SHSs^(19,20)^. The relationship between BMI and education has already been confirmed in other studies^(37–39)^.

In the present study, smoking was associated with lower BMI in women. In the literature, smoking is often associated with lower BMI^(40,41)^, however heavy smoking has been suggested to be associated with higher BMI. The direction of causality is still unclear, it is possible that individuals with high BMI smoke to reduce weight^(42)^. Unfortunately, in the present study, we did not have information on the level of tobacco use and cannot investigate the relationship in more detail.

For the first time, different studies were synthesised to show the association between lifestyle factors as risk factors and increased BMI in Switzerland. For instance, a higher BMI was not associated with the intake of fruits and vegetables, milk and dairy or fish but was associated with meat and sweetened beverage consumption. This shows that the intake of an unhealthy diet seems to have a stronger impact on BMI when compared to the intake of a healthy diet. A meta-analysis also confirmed that less consumption of red and processed meat was associated with lower BMI^(43)^. One reason might be that greater animal protein consumption is related to weight gain^(44)^. This can be relevant when developing overweight preventive strategies. For other factors, it is important to consider a possible reversed causality, such as for healthy sleep or for self-rated health. It is possible that people who are pre-obese or obese have poorer sleep and rate their health more poorly but could also be at an increased risk for obesity^(45)^. As all surveys included in the present analysis are cross-sectional, it is not possible to exclude either possibility.

Another interesting finding was the association between height and BMI. We found that the probability of having a higher BMI decreased with being taller, and the AIC analysis showed that body height was the most important variable explaining BMI, after age and education. Similar findings were reported in the literature: A study from Portugal showed a decreasing mean height with increasing BMI categories^(46)^. Another study carried out in 25 diverse populations from the US, Europe and Asia showed that BMI was not independent of height and that the relationship between weight and height differed significantly between males and females^(47)^. Adult height cannot be changed. However, growth is subject to several physiological, environmental and socio-economic factors that can be addressed in public health efforts for the prevention of obesity and other health outcomes. The mechanisms behind the association between height and BMI are not entirely understood at the moment. The relationship between body height and BMI could be affected by other variables. Even if we adjust the models for educational level, it is possible that residual confounding by socio-economic background still plays some role^(48)^. Moreover, aspects of the development of body proportions and metabolic traits may contribute to it^(49,50)^. Our study has strengths and limitations. One strength is that, to our knowledge, this is the first synthesis study on excess and underweight in Switzerland including eight nationwide studies. Some results are novel and could not be deduced from single studies. A limitation was that not all studies included all variables under examination; therefore, certain subanalyses were restricted to data from a subset of datasets. The surveys included were from different years, but recent studies have shown that the average weight of Swiss schoolchildren and Swiss conscripts has remained stable since 2010^(12,13)^. Moreover, the distribution of BMI is very similar between the SHS 2012 and 2017, as well as between the SFP 2010 and 2017. The same is true for the explanatory variables. There are hardly any differences in the distributions between the years, except for the variable ‘Urbanity’. In 2017, slightly more participants in the SFP live in urban areas than in 2012. Therefore, we believe that the different survey years did not have a major impact on this study. Another limitation was that the surveys used different methodologies for sampling and for data collection. We mitigated some of these differences by using *z*-scores for some variables and by including the eight discrete studies as random factors to control for variance within and between the eight discrete studies in our models. However, we cannot exclude some remaining differences between the datasets. The cross-sectional nature of the data limited causal inference. Our models do not consider interactions between socio-demographic and lifestyle factors, which may provide additional insights, as it has been shown that people with overweight do not constitute a homogeneous group and that lifestyle factors might play a particularly strong role in certain socio-demographic subgroups.

## Conclusion

The present study was the first to synthesise eight studies to show the association between several lifestyle factors as risk factors and underweight, pre-obesity and obesity in Switzerland.

Our results confirmed that socio-demographic factors were among the strongest determinants of BMI, along with lifestyle factors such as diet, exercise, smoking or sleep. Lifestyle factors are important for public health interventions. For example, a higher BMI was particularly associated with increased consumption of meat and sweetened beverages. This finding may indicate that special attention can be paid to reducing the intake of an unhealthy diet as part of obesity prevention strategies. However, further research is needed to determine whether lifestyle factors are particularly important for explaining excess and underweight in certain subgroups of the population.

## Supplementary material

For supplementary material accompanying this paper visit https://doi.org/10.1017/jns.2022.66. click here to view supplementary material
