# Supplementary material for: A quantitative synthesis study on body mass index and associated factors among adult men and women in Switzerland
Source: J Nutr Sci. 2022 Aug 10;11:e65. doi: 10.1017/jns.2022.66 (PMC9379928; doi:10.1017/jns.2022.66)
Supplement: Supplementary file 1 [file S2048679022000660sup001.pdf]

Supplementary Table 1: Δ AIC for each independent variable. The higher Δ AIC is, the more important the variable. The three most important variables are each marked in green. A negative value indicates an unimportant variable.

Variables	Models (studies included)												
	Basis model (8)	Smoking (5)	Alcohol consumption (5)	Fruit & Vegetable consumption (7)	Self-rated health status (7)	Urbanity (5)	Nationality (7)	Meat consumption (6)	Fish consumption (6)	Milk & dairy consumption (4)	Sweetened beverage consumption (4)	Following recommendation of physical activity (3)	Sleep disorder (2)
**Men**													
Age	997.32	740.16	701.29	951.35	579.86	699.76	1075.45	754.87	749.54	394.32	419.02	260.81	494.70
Education	184.12	100.81	135.75	170.68	130.16	133.49	165.8	137.06	135.79	64.24	105.44	50.56	47.42
Body height	48.77	27.21	41.74	48.73	40.37	40.93	41.31	40.03	38.76	32.74	49.29	12.82	29.74
Physical Activity	22.81	-3.27	22.14	18.42	12.86	22.97	6.32	26.93	26.79	24.98	28.81		-4.73
Language region	-2.69	-4.50	-3.79	-3.76	-4.06	-2.67	1.23	-1.64	-2.15	-1.85	-2.25	-5.62	-3.56
Smoking		-3.88											
Alcohol consumption			-2.53										
Fruit & Vegetable consumption				-3.36									
Self-rated health status					695.37								
Urbanity						19.67							
Nationality							76.38						
Meat consumption								21.22					
Fish consumption									0.14				
Milk & dairy consumption										-2.70			
Sweetened beverage consumption											18.08		
Following recommendation of physical activity												72.77	
Sleep disorder													55.52
**Women**													
Age	915.23	649.05	653.38	834.49	605.72	653.39	1075.45	692.18	675.24	437.1	308.27	239.94	498.08
Education	624.43	428.50	500.81	594.68	525.75	497.78	165.80	494.53	509.14	294.37	307.24	133.51	244.89
Body height	285.25	200.77	213.69	272.14	250.06	215.56	41.31	219.74	216.75	94.0	141.66	54.96	122.48
Physical Activity	31.50	-1.72	34.69	33.25	16.66	34.17	6.32	38.15	41.68	62.02	34.48		-5.42
Language region	56.12	31.95	30.96	56.73	59.28	26.05	1.23	32.41	33.19	21.41	4.45	11.89	15.36
Smoking		13.29											
Alcohol consumption			3.79										
Fruit & Vegetable consumption				-4.53									
Self-rated health status					860.9								
Urbanity						11.51							
Nationality							76.38						
Meat consumption								63.59					
Fish consumption									-1.58				
Milk & dairy consumption										-2.14			
Sweetened beverage consumption											11.94		
Following recommendation of physical activity												51.77	
Sleep disorder													26.54
